# Entropy in Image Analysis

**DOI:** 10.3390/e21050502

**Published:** 2019-05-17

**Authors:** Amelia Carolina Sparavigna

**Affiliations:** Department of Applied Science and Technology, Polytechnic University of Turin, 10129 Turin, Italy; amelia.sparavigna@polito.it

**Keywords:** image entropy, Shannon entropy, generalized entropies, image processing, image segmentation, medical imaging, remote sensing, security

Image analysis is playing a very essential role in numerous research areas in the fields of science and technology, ranging from medical imaging to the computer science of automatic vision. Being involved in several applications, which are mainly based on a constant innovation of technologies, image analysis always requires different approaches and new algorithms, including continual upgrades and improvements of existing methods. Accordingly, the range of the analyses in which it is engaged can be considered as wide as the prospective future technologies.

A challenge of image analysis is obtaining meaningful information by extracting specific features from images contained in large databases or during real-time acquisition. Among the problems requiring feature extraction are, to name a few, face detection and recognition, character recognition, and parametric determinations for augmented reality and other technologies. Another challenge is the secure encryption and decryption of multimedia data. These tasks demand highly sophisticated numerical and analytical methods.

The contributions to this Special Issue provide a good overview of the most important demands and solutions concerning the abovementioned extraction, encryption, and decryption of data. In all the contributions, entropy plays a pivotal role. In the following, the reader can find subjects and problems according to the order of their publication.

Lu et al. [1] consider a method for establishing an automatic and efficient image retrieval system. The proposed solution is an adaptive weighting method based on entropy and relevance feedback. Among the advantages of the proposed solution, an improved retrieval ability and accuracy of feature extraction are featured.

Zhu et al. investigate image data security in [2]. Image encryption is necessary to protect digital image transmission. The method proposed in the article is based on chaos and Secure Hash Algorithm 256 (SHA-256). Experimental results were used to check the algorithm, showing that it is safe and reliable.

Saqib and Kazmi [3] propose a solution to the problem of the retrieval and delivery of contents from audio-video repositories, in order to achieve faster browsing of collections. The compression of data is achieved by means of keyframes, which are representative frames of the salient features of the videos.

Karawia [4] reports an encryption algorithm for image data security to protect the transmission of multiple images. The algorithm is based on the combination of mixed image elements (MIES) and a two-dimensional economic map. Pure image elements (PIES) are used. The analysis of the experimental results verifies the proposed algorithm as efficient and secure.

A chaos-based image encryption scheme is the subject of an improved cryptanalysis proposed in [5] by Zhu et al. Their analysis integrates permutation, diffusion, and linear transformation processes. A color image encryption scheme is also given. Experimental results and a security analysis of the proposed cryptosystem are provided as well. 

As pointed out in Yang et al. [6], distortions are usually introduced in images by their acquisition, and by their compression, transmission, and storage. An image quality assessment (IQA) method is therefore required. In their contribution, the authors propose an effective blind IQA approach for natural scenes and validate its performance.

Lin et al. [7] investigate a problem of medical analysis, concerning ultrasound entropy imaging. This imaging is compared with acoustic structure quantification (ASQ), a typical method for analyzing backscattered statistics. To illustrate this analysis, they describe a case study on the fat accumulation in the liver.

As stressed in Li et al. [8], it is not possible to capture all the details of a scene by means of a single exposure. Multi-exposure image fusion is required. In the algorithm proposed by the authors, the image texture entropy has its most relevant role in the adaptive selection of image patch sizes.

Image encryption returns in [9], where Huang and Ye propose an encryption algorithm based on a chaotic map. The two-dimensional chaotic map is the 2D Sine Logistic Modulation Map (2D-SLMM). The sophisticated use of keystream, time delay, and diffusion gives a high sensitivity to keys and plain images. 

Today, the classification of hyperspectral images, those currently used for mapping the state of the Earth’s surface, is fundamental. Consequently, approaches to characterize the quality of classified maps are required. Shadman Roodposhti et al. [10] discuss the uncertainty assessment of the emerging classification methods.

Mejia et al. [11] consider one of the fundamental tools of medical imaging. It is the imaging technique based on the reconstruction of positron emission tomography (PET) data. The authors propose a method that includes models of a priori structures to capture anatomical spatial dependencies of the PET images.

In this Special Issue, research devoted to the study of the surface quality of 3D printed objects is highlighted. An application of this is proposed by Fastowicz et al. [12]. The method is based on the analysis of the surface regularity during the printing process. In the case of the detection of low quality, some corrections can be made or the printing process aborted.

In Li et al. [13], a new approach to the registration of images is described. The method is based on Arimoto entropy with gradient distributions. The proposed approach provides a nonrigid alignment, based on an optimal solution of a cost function.

Miao et al. propose, in [14], a method for evaluating the anti-skid performance of asphalt pavement surfaces. Three-dimensional macro- and micro-textures of asphalt surfaces are detected. The method based on entropy is compared to the traditional macrotexture parameter Mean Texture Depth index.

Mello Román et al. [15] report a processing that improves the details of infrared images in [15]. The method aims to enhance contrast. At the same time, it preserves the natural appearance of images. A multiscale top-hat transform is used.

The encryption of images is also a hot topic of this Special Issue. Wen et al. [16] present another relevant work on this subject. Their paper illustrates a study of the image encryption algorithm based on DNA encoding and spatiotemporal chaos (IEA-DESC). It is shown that the IEA-DESC algorithm has some inherent security problems that need a careful check.

Nagy et al., in their article concerning the imaging of colonoscopy [17], propose a research with its framework in the methods based on the structural Rényi entropy. The aim of their work is to contribute to computer-aided diagnoses in finding colorectal polyps. The authors investigate characteristic curves that can be used to distinguish polyps and other structures in colonoscopy images.

Information entropy is involved in binary images and primality, as shown by an article in the Special Issue which deals with the hidden structure of prime numbers [18]. As demonstrated by the author, Emanuel Guariglia, the construction of binary images enables the generalization of numerical studies, which have indicated a fractal-like behavior of the prime-indexed primes (PIPs). PIPs are compared to Ramanujan primes to investigate their fractal-like behavior as well.

In Lang and Jia [19], the Kapur entropy for a color image segmentation is discussed. A new hybrid whale optimization algorithm (WOA), possessing a differential evolution (DE) as a local search strategy, is proposed to better balance the exploitation and exploration phases of optimization. Experimental results of the WOA-DE algorithm are proposed.

Li et al. [20] address image encryption by means of a method that integrates a hyperchaotic system, pixel-level Dynamic Filtering, DNA computing, and operations on 3D Latin Cubes—namely, a DFDLC image encryption. Experiments show that the proposed DFDLC encryption can achieve state-of-the-art results.

The problem of the multilevel thresholding segmentation of color images is considered in the work of Song et al. [21], according to a method based on a chaotic Electromagnetic Field Optimization (EFO) algorithm. The entropy involved in the method is fuzzy entropy. The EFO algorithm is a process inspired by the electromagnetic theory developed in physics.

The q-sigmoid functions, based on non-extensive Tsallis statistics, appear in [22]. Sergio Rodrigues et al. use them to enhance the regions of interest in digital images. The potential of q-sigmoid is demonstrated in the task of enhancing regions in ultrasound images, which are highly affected by speckle noise.

This Special Issue ends with a work devoted to an image processing method for person re-identification [23]. The method proposed by Ma et al. is based on a new deep hash learning, which is an improvement on the conventional method. Experiments show that the proposed method has comparable performances or outperforms other hashing methods.

As we have seen from the short descriptions of its contributions, this Special Issue shows that entropy in image analysis can have several variegated applications. However, applications of entropy are not limited to those described here. For this reason, the Guest Editor hopes that the readers, besides enjoying the present works, can receive positive hints from the reading and fruitful inspirations for future research and publications.
